# Unlike Pancreatic Cancer Cells Pancreatic Cancer Associated Fibroblasts Display Minimal Gene Induction after 5-Aza-2′-Deoxycytidine

**DOI:** 10.1371/journal.pone.0043456

**Published:** 2012-09-11

**Authors:** Jun Yu, Kimberly Walter, Noriyuki Omura, Seung-Mo Hong, Angela Young, Ang Li, Audrey Vincent, Michael Goggins

**Affiliations:** 1 Department of Pathology, The Sol Goldman Pancreatic Cancer Research Center, The John Hopkins Medical Institutions, Baltimore, Maryland, United States of America; 2 Department of Medicine, The Sol Goldman Pancreatic Cancer Research Center, The John Hopkins Medical Institutions, Baltimore, Maryland, United States of America; 3 Department of Oncology, The Sol Goldman Pancreatic Cancer Research Center, The John Hopkins Medical Institutions, Baltimore, Maryland, United States of America; Geisel School of Medicine at Dartmouth, United States of America

## Abstract

**Purpose:**

Cancer associated stromal fibroblasts (CAFs) undergo transcriptional and phenotypic changes that contribute to tumor progression, but the mechanisms responsible for these changes are not well understood. Aberrant DNA methylation is an important cause of transcriptional alterations in cancer cells but it is not known how important DNA methylation alterations are to CAF behavior.

**Experimental Design:**

We used Affymetrix exon arrays to compare genes induced by the DNA methylation inhibitor 5-aza-dC in cultured pancreatic cancer associated fibroblasts, pancreatic control fibroblasts and pancreatic cancer cell lines.

**Results:**

We found that pancreatic CAFs and control pancreatic fibroblasts were less responsive to 5-aza-dC-mediated gene reactivation than pancreatic cancer cells (mean+/−SD of genes induced ≥5-fold was 9±10 genes in 10 pancreatic CAF cultures, 17±14 genes in 3 control pancreatic fibroblast cultures, and 134±85 genes in 4 pancreatic cancer cell lines). We examined differentially expressed genes between CAFs and control fibroblasts for candidate methylated genes and identified the disintegrin and metalloprotease, *ADAM12* as hypomethylated and overexpressed in pancreatic CAF lines and overexpressed in fibroblasts adjacent to primary pancreatic adenocarcinomas.

**Conclusions:**

Compared to pancreatic cancer cells, few genes are reactivated by DNMT1 inhibition in pancreatic CAFs suggesting these cells do not harbor many functionally important alterations in DNA methylation. CAFs may also not be very responsive to therapeutic targeting with DNA methylation inhibitors.

## Introduction

Epigenetic changes in gene expression are a fundamental feature of cancer cells [1]. One of the best characterized epigenetic alterations is DNA methylation. DNA methylation patterns are heritable and are maintained after cell division mainly by the DNA methyltransferase Dnmt1. Aberrant DNA hypermethylation often occurs at CpG islands in gene promoters and is associated with a closed chromatin state and gene repression. Considerable evidence indicates that aberrant hypermethylation and gene silencing of tumor suppressors and other regulatory genes contributes to the development of pancreatic and other cancers [2]. Aberrant hypomethylation of normally methylated and silenced genes has also been described in pancreatic and other cancers as a cause of aberrant gene overexpression [3], [4].

The mechanisms responsible for these aberrant methylation patterns in cancers are not well understood, but suspected mechanisms include overexpression of *DNMT1*, accumulation of DNA methylation alterations with age [5], [6] and environmental influences [7], altered activity of the *de novo* methylating enzymes *DNMT3a* and *DNMT3b* [8], [9], and possibly from altered cellular microenvironment such as from chronic inflammation [3], [10].

Non-neoplastic stromal cells within the tumor microenvironment also undergo transcriptional and other phenotypic changes relative to normal cells that are thought to contribute to tumor progression. Pancreatic cancer is a deadly disease [11] and is well known for its extensive desmoplastic stromal response comprising an average 75% of the cells in the tumor mass [12]. It is suspected that the stromal component contributes to the resistance of pancreatic cancers to drug therapies [13], and several studies implicate differences in stromal behavior with patient outcome in pancreatic and other cancers [14], [15], [16], [17], [18]. Cancer associated fibroblasts (CAFs), the predominant cell type in the stromal microenvironment, adopt an activated phenotype during tumorigenesis, undergoing morphologic, functional, and gene expression changes relative to normal fibroblasts [19]. These activated CAFs are characterized by α-smooth-muscle actin (α-SMA) expression [20], enhanced contractile and secretory ability, and increased synthesis of collagens, extracellular matrix proteins [21] and growth factors including epithelial growth factor, platelet-derived growth factors (PDGF), basic fibroblast growth factor, hepatocyte growth factor, and transforming growth factor beta (TGF-β) [22]. Through these and other signals, CAFs interact intimately with tumor cells to promote their growth, and therefore CAFs are of considerable interest as a therapeutic target. Indeed, recent strategies to target CAFs include the use of the kinase inhibitor, imatinib to block stromal PDGF receptors [23], antibodies to block vascular endothelial growth factor (VEGF) derived from both cancer cells and CAFs to inhibit tumor angiogenesis [24] and the use of smoothened (Smo) inhibitors to block tumor-stromal Hedgehog signaling and deplete stromal fibroblasts that overexpress the hedgehog (Hh) receptor Smo [25], [26], [27], [28]. That initial trials of smoothened inhibitors in pancreatic cancers did not show evidence of benefit highlights the need for basic studies investigating tumor stromal interactions. Another potential benefit of targeting cancer stroma is to improve the accessibility of therapeutics to pancreatic cancer cells. For example, the drug nab-paclitaxel (Abraxane), a nanoparticle formulation of paclitaxel, binds to Sparc, a stromal matrix protein often highly expressed in pancreatic cancer stroma that mediates tumor stromal interactions, thereby potentially improving drug delivery to the tumor [29], [30]. Clinical trials of abraxane in pancreatic cancer show promise and in animal models there is evidence to suggest that the delivery of gemcitabine to the tumor is improved with abraxane, and with hedgehog inhibitors [25]. Efficacy of the Gemcitabine/abraxane drug combination still awaits the results of phase 3 clinical trials.

The influence of CAFs on cancer growth has been demonstrated in numerous studies [31], [32] but the molecular mechanisms underlying the CAF phenotype are not well understood. Although a major influence of CAFs is suspected to be the tumor microenvironment including influences from the cancer cells themselves, CAFs retain their tumor-promoting properties *in vitro* after prolonged cell culture [33], suggesting that hereditary mechanisms are responsible. Although genetic alterations in CAFs have been described, more recent studies investigating the possibility that CAFs undergo clonal genetic alterations similar to cancer cells have found no evidence for such alterations [33], [34], [35], [36], [37], [38]. In the absence of widespread genetic mutations, it is reasonable to suspect that epigenetic mechanisms such as DNA methylation, which are mitotically heritable, are responsible for the stable gene expression changes in CAFs. Indeed, several genes implicated in tumor-stromal interactions and induced in CAFs by coculture with cancer cells, such as *SPARC* [29], [30], *COX-2* and matrix-metalloproteinases (MMPs) [39] are regulated by DNA methylation. Furthermore, CAFs are subjected to the same influences in the tumor microenvironment which are thought to contribute to methylation changes in cancer cells, such as chronic inflammation [34].

The heterogeneity of CAFs from different tumors and even within the same tumor [14], [30] also suggests that CAFs have multiple sources which could contribute to epigenetic differences. In addition to activation of resident fibroblasts in the tumor microenvironment, some CAFs are bone-marrow derived mesenchymal precursor cells [40], [41] and perhaps epithelial or endothelial cells undergoing mesenchymal transition [21], [42]. Because these differentiation and transdifferentiation processes are regulated by epigenetic mechanisms [43], CAFs derived from different sources have different epigenetic and transcriptional profiles compared to their resident tissue counterparts.

Only a few studies have begun to explore epigenetic mechanisms for these transcriptional changes in CAFs. One of the first studies to provide evidence of DNA methylation differences between normal and tumor associated stromal cells took a genome-wide approach using methylation specific digital karyotyping (MSDK) to profile epithelial cells, myoepithelial cells and stromal fibroblasts during breast tumor progression [35]. Another approach combining laser capture microdissection with methylation specific PCR (MSP) of candidate genes identified frequent promoter methylation of *GSTP1* and *RARB2* in prostate tumor-associated stromal cells relative to normal prostate stromal cells [44]. A third approach using methylation-sensitive SNP array analysis (MSNP) demonstrated focal gains in methylation and global hypomethylation in gastric cancer-associated myofibroblasts [36].

One useful strategy for identifying methylation events is to perform a gene reactivation screen using DNA methylation inhibitors such as 5-aza-2′-deoxycytidine (5-aza-dC), which selectively targets the DNMT1 enzyme for depletion. This method has the advantage of identifying the DNA methylation alterations that regulate transcription and therefore more likely to be functionally important. Although this approach has successfully identified methylation events in cancer cells from multiple tumor types [45] to our knowledge this approach has not been used to identify genes regulated by methylation in CAFs. To determine whether DNA methylation are similarly important regulators of gene expression changes in CAFs as for cancer cell lines, we performed a genome-wide reexpression analysis of human pancreatic cancer-associated fibroblasts and pancreatic cancer cell lines using 5-aza-dC.

## Materials and Methods

### Culture of Cell lines

Primary cultures of stromal fibroblasts, designated cancer associated fibroblasts (CAFs) CAF9, CAF11, CAF12, CAF13, CAF14, CAF15, CAF16, CAF18, CAF19, CAF20, CAF21, CAF22, CAF25, and CAF35 were established from surgically-resected pancreatic cancer tissue from 13 patients (6 males mean ± standard deviation (SD) age of 58±7 years) with clinically sporadic pancreatic ductal adenocarcinoma. The cancers were all moderate to poorly differentiated with a mean tumor size of 3.5 cm. These primary CAF cultures were established as previously described [27] as were two hTERT-immortalized control fibroblasts (SC2 and SC3, established from surgically resected non-neoplastic pancreas tissue from 2 females (mean age, 63 years) [27]. The immortalized human pancreatic Nestin-expressing (HPNE) cell line was generously provided by Dr. Michel Ouellette (University of Nebraska Medical Center) [46]. Four pancreatic cancer cell lines, including A32-1, Panc2.8, Panc3.014 and Panc215 established from primary pancreatic ductal adenocarcinomas at our institution and described previously [47] were also used in this study. All cell lines were maintained in DMEM (4.5 mg/mL glucose; Invitrogen) containing 10% FBS under standard conditions as described previously [48]. All studies were performed with approval from the Johns Hopkins Committee for Clinical Investigation.

### 5-aza-dC treatment and RNA extraction

Cells were plated at 2×10^5^ cells per T75 flask and treated the following day with 1 µmol/L 5-aza-2′-deoxycytidine (5-aza-dC, Sigma) [49] for 4 days during the exponential growth phase, with a change of media and drug every 24 hours. On day 5 when cells were near-confluent, they were washed with cold PBS and total RNA isolated using a Qiagen kit (Qiagen) or the mirVana miRNA kit (Ambion), according to manufacturer's instructions as described previously [50].

### Exon array and selection of genes for validation

The Affymetrix GeneChip Human Exon 1.0ST Array platform was used to analyze gene expression patterns in untreated and 5-aza-dC treated fibroblasts and cancer cell lines, as previously described [27]. We are in compliance with the Minimum Information about a Microarray Experiment guidelines and have submitted our microarray data set to the Gene Expression Omnibus repository (GEO Accession #GSE20911).

### Quantitative RT-PCR (qRT-PCR)

1 µg total RNA was reverse transcribed according to the manufacturer's instructions (Invitrogen). The resulting cDNA was amplified on an ABI 7300 Real-Time PCR thermocycler using SYBR GREEN PCR Master Mix and recommended PCR conditions (Applied Biosystems). The housekeeping gene *GAPDH* was used for normalization. Primer specificity was confirmed by melting curve analysis. Results are expressed as normalized expression values relative to the indicated cell line ( = 2^−ΔΔCt^). All PCR reactions were performed in triplicate. Primers sequences are as follows: *Stratifin* RTsense: 5′-TCTGATCCAGAAGGCCAAG-3′; *Stratifin* RTantisense: 5′-GTTTCGCTCTTCGCAGGAG-3′; *TKTL1* RTsense: 5′-AGCTCCGGCCACCCTACATCATG-3′; *TKTL1* RTantisense: 5′-TGCCACATCCACAAACGACAGTCT-3′; *ADAM12* RTsense: 5′-AAATGAAGGTCTCATTGCCAG-3′; *ADAM12* RTantisense: 5′-AGAATTACCCGTGTAATTTCGAG-3′; *GAPDH* RTsense: 5′-CAACAGCCTCAAGATCATCAG-3′; *GAPDH* RTantisense: 5′-ACTGTGGTCATGAGTCCTTC-3′.

### Bisulfite Sequencing

The methylation status of the 5′ CpG islands of candidate genes was determined by bisulfite sequencing. Bisulfite modification and PCR was performed as previously described [51]. Bisulfite Modified Sequencing (BMS) primers were as follows: *TKTL1* BMS forward: 5′-TGTGTAGAGAAAGAAGATTTTGTATT-3′, *TKTL1* BMS reverse: 5′-CCCTTTAAAATCTAAAAACCCACTC-3′, and internal sequencing primer *TKTL1* BMS-Seq: 5′-GATTGTAGGAGAGAAGATGAG-3′; *ADAM12* BMS forward: 5′-TTTAGTTTTAGTTTGAAAAGTTGGA-3′, *ADAM12* BMS reverse: 5′-CTAAACTCTTCTAACCTTTCAT-3′, and internal sequencing primer *ADAM12* BMS-Seq: 5′-TTCTAACACAAACCAACCTTAACC-3′. Purified products were sequenced at the Johns Hopkins Core Sequencing Facility.

### Western Blot Analysis of DNMT1 Expression

Western blot analysis was performed using lysates from untreated or 5-aza-dC-treated HPNE or CAF12 cells. A Bradford assay was used to estimate protein concentration, using bovine serum albumin (BSA, Invitrogen) as a standard. Equal amounts of protein (40 µg per lane) were separated by 4–12% gradient SDS gel electrophoresis (Invitrogen), transferred to nitrocellulose membranes, and incubated in blocking solution (5% milk in TBS with 0.1% Tween 20) for 30 minutes. Membranes were incubated for 60 minutes with an anti-DNMT1 polyclonal antibody (generously provided by Dr. Bill Nelson, JHU) at 1∶200 dilution or a monoclonal antibody against GAPDH (Cell Signaling, Danvers, MA) at 1∶2000 dilution. Secondary HRP-linked antibodies (Santa Cruz Biotechnology, and Amersham Biosciences) were applied at 1∶2000 dilution and proteins detected using an ECL kit (Amersham Biosciences).

### Pancreatic Adenocarcinoma Tissue Microarrays and Adam12 Immunohistochemistry

The expression of Adam12 protein was examined utilizing immunohistochemical (IHC) labeling of formalin-fixed, paraffin-embedded tissue microarrays (TMAs) using a DAKO Autostainer (DAKO, Carpinteria, CA). Four TMAs containing a total of 72 different surgically resected pancreatic ductal adenocarcinomas and corresponding normal pancreas tissues were constructed as previously described [29]. Adam12 IHC staining was performed as previously described [29] using a rabbit anti-human Adam12 antibody (A2601, Sigma) at a 1∶100 dilution and a 60 minute incubation time. Labeling was performed using the Envision-sPlus Detection Kit (DAKO).

### Statistical Analysis

Descriptive statistical values and plots were generated using Microsoft Excel package or the Partek® Genomics Suite™ v6.3 beta. The Robust Multichip Average (RMA) method was used to normalize the raw intensity measurements of all probe sets. Gene expression values were then obtained using the one-step Tukey's biweight method. Two-way ANOVA was done to identify significant expression changes between untreated and 5-aza-dC-treated fibroblasts or cancer cell lines, or between CAFs and control fibroblasts. Differences were considered significant at *P*<0.05, and values reported are means ± SD.

## Results

### Global gene expression analysis of human pancreatic fibroblasts and cancer cell lines treated with 5-aza-dC

We established primary pancreatic CAF cultures and control fibroblast cultures as previously described [38]. Using global gene expression profiling, we previously identified gene expression differences in pancreatic CAFs relative to control fibroblasts [27]. Hierarchical clustering of the gene expression profiles of untreated CAF and control fibroblasts is provided in Figure S1. Suspecting that these changes in gene expression were in part mediated by changes in DNA methylation, we compared the gene expression profiles of CAF and control fibroblast cultures before and after treatment with 5-aza-dC using Affymetrix Exon Arrays (ST 1.0) (CAF11, CAF12, CAF13, CAF15, CAF16, CAF18, CAF19, CAF22, CAF25, and CAF35 for CAFs; HPNE, SC2, and SC3 for control fibroblast cells, respectively).

**Figure 1:**
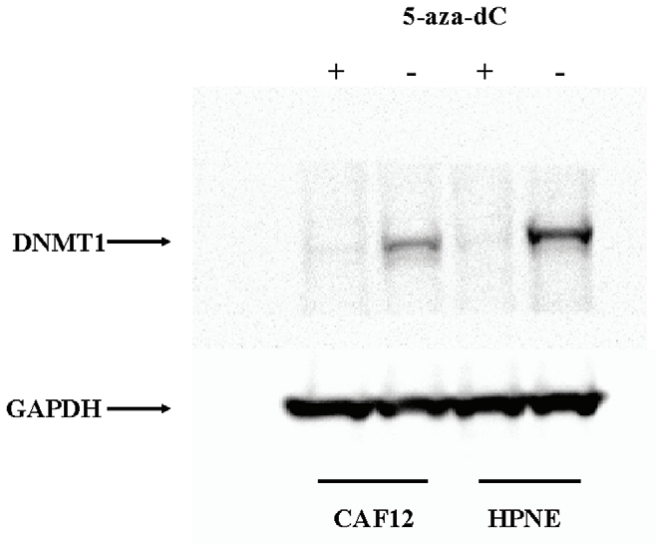
Effect of 5-aza-dC treatment on DNMT1 protein levels by Western blot analysis of pancreatic CAF and control fibroblast cultures. DNMT1 protein is depleted (relative to GAPDH) in 5-aza-dC treated HPNE and CAF12 cells.

We first confirmed 5-aza-dC depletion of the Dnmt1 enzyme by Western blot. 5-aza-dC treatment of HPNE and CAF12 cells resulted in depletion of Dnmt1 protein at 1 µM concentrations but not GAPDH control protein (Figure 1). To further examine the effect of 5-aza-dC concentrations, we compared the number of genes induced by 1 µM and 10 µM of 5-aza-dC in two additional CAFs, CAF19 and CAF35, and found no major difference in the number of genes induced between these drug concentrations (data not shown). We therefore used 1 µM concentrations of 5-aza-dC to treat each CAF. We treated CAF cells for 4 days as this was sufficient duration for cell proliferation. Longer treatments (for 5–7 days) often resulted in CAFs growing to confluence (data not shown). Consistent with this, we performed proliferation assays on CAF19 at different 5-aza-dC concentrations (0 µM, 1 µM, 5 µM, 10 µM, and 20 µM) and found that growth was not significantly affected at the 1 µM concentration we employed (Figure S2).

After comparing the expression profiles of each CAF before and after 5-aza-dC treatment, we first identified genes that were upregulated by an overall fold-change average of ≥2.0 in all ten 5-aza-dC-treated CAFs relative to untreated CAFs. We chose a 2-fold cut-off as more modest differences in RNA were considered more likely to be related to experimental variation. Since we were interested in identifying genes silenced in CAFs whose expression was induced by 5-aza-dC, we then excluded the subset of genes that were expressed in untreated CAFs. This criterion identified 42 candidate genes (Table 1).

**Table 1:** Genes upregulated by an overall fold change of ≥2.0 in ten 5-aza-dC-treated pancreatic CAFs relative to untreated CAFs.

Gene symbol	Gene Assignment	*P* value	Average Fold Change	Upregulated in 1 or more control fibroblasts
DAZL	NM_001351//deleted in azoospermia-like	0.016	11.4	Yes
SPANXB1	NM_032461//SPANX family, member B1	0.296	8.9	Yes
GTSF1	NM_144594//gametocyte specific factor 1	0.047	6.4	Yes
MT1G	NM_005950//metallothionein 1G	0.022	4.5	Yes
MAGEB2	NM_002364//melanoma antigen family B, 2	0.055	4.3	Yes
ASB5	NM_080874//ankyrin repeat and SOCS box-containing 5	0.048	3.3	Yes
HAPLN1	NM_001884//hyaluronan and proteoglycan link protein 1	0.15	3.3	Yes
MYH3	NM_002470//myosin, heavy chain 3, skeletal muscle, embryonic	0.062	3.1	No
IL18	NM_001562//interleukin 18 (interferon-gamma-inducing factor)	0.064	3.1	Yes
HIST1H1T	NM_005323//histone cluster 1, H1t	0.071	3	Yes
TKTL1	NM_012253//transketolase-like 1	0.034	2.9	Yes
KRT81	NM_002281//keratin 81	0.03	2.8	Yes
MT1H	NM_005951//metallothionein 1H	0.067	2.8	Yes
TRIM55	NM_033058//tripartite motif-containing 55	0.119	2.7	Yes
LAPTM5	NM_006762//lysosomal protein transmembrane 5	0.011	2.7	Yes
H2AFB1	NM_001017990//H2A histone family, member B1	0.107	2.6	Yes
NXPH2	NM_007226//neurexophilin 2	0.006	2.6	No
LCE2D	NM_178430//late cornified envelope 2D	0.366	2.5	Yes
ANXA3	NM_005139//annexin A3	0.005	2.5	Yes
OBP2B	NM_014581//odorant binding protein 2B	0.542	2.4	Yes
SPANXE	NM_145665//SPANX family, member E	0.142	2.4	Yes
UPK1B	NM_006952//uroplakin 1B	0.037	2.4	Yes
PRY	NM_004676//PTPN13-like, Y-linked	0.581	2.4	Yes
MAGEA4	NM_001011548//melanoma antigen family A, 4	0.042	2.4	Yes
SUSD2	NM_019601//sushi domain containing 2	0.047	2.3	Yes
MYL7	NM_021223//myosin, light chain 7, regulatory	0.04	2.3	No
SFN	NM_006142//stratifin	0.06	2.2	Yes
IL20RB	NM_144717//interleukin 20 receptor beta	0.205	2.2	Yes
AQP1	NM_198098//aquaporin 1 (Colton blood group)	0.024	2.2	Yes
MAEL	NM_032858//maelstrom homolog (Drosophila)	0.052	2.2	Yes
IFI27	NM_001130080//interferon, alpha-inducible protein 27	0.014	2.2	Yes
ACTC1	NM_005159//actin, alpha, cardiac muscle 1	0.022	2.2	No
TRIML2	NM_173553//tripartite motif family-like 2	0.042	2.2	No
VAMP8	NM_003761//vesicle-associated membrane protein 8 (endobrevin)	0.016	2.1	Yes
TMEM92	NM_153229//transmembrane protein 92	0.049	2.1	Yes
KRT14	NM_000526//keratin 14	0.11	2.1	Yes
KRTAP13-4	NM_181600//keratin associated protein 13-4	0.22	2.1	No
ANKRD1	NM_014391//ankyrin repeat domain 1 (cardiac muscle)	0.197	2.1	Yes
TYROBP	NM_003332//TYRO protein tyrosine kinase binding protein	0.067	2.1	Yes
TUBA4A	NM_006000//tubulin, alpha 4a	0.1	2	Yes
KRT17	NM_000422//keratin 17	0.051	2	No
LYPD1	NM_144586//LY6/PLAUR domain containing 1	0.001	2	Yes

**Figure 2:**
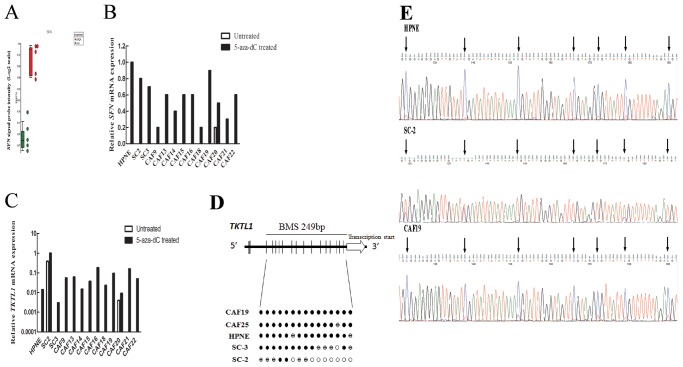
Effect of 5-aza-dC treatment on *SFN* and *TKTL1* mRNA expression in 5-aza-dC treated pancreatic CAFs and control fibroblasts, and bisulfite sequencing analysis of *TKTL1*. (A) Affymetrix exon array analysis of *SFN* mRNA expression in five pancreatic CAFs before (green) and after (red) 5-aza-dC treatment. (B) and (C) Quantitative RT-PCR analysis of *SFN* and *TKTL1* mRNA expression relative to *GAPDH* mRNA in pancreatic fibroblast cultures before (blue bars) and after (red bars) 5-aza-dC treatment. Each assay was performed in triplicate. Data are means of three independent experiments; bars are SD values. (D) Top: *TKTL1* gene structure and distribution of CpG dinucleotides. Short vertical bars represent CpG sites. Arrow points to transcriptional start site. Below: Bisulfite genomic sequencing analysis in pancreatic CAFs and control fibroblasts. Open circles represent unmethylated CpG sites, solid black circles methylated CpG sites, and hatched circles partially methylated CpG sites. (E) Bisulfite sequencing chromatograms of the *TKTL1* promoter in a pancreatic CAF (CAF19) and control fibroblast lines (HPNE and SC2). Arrows point to cytosine residues.

To further confirm the 5-aza-dC mediated gene induction we performed qRT-PCR on the gene *Stratifin* (14-3-3 sigma) and *Transketolase-like protein 1* (*TKTL1*) because their expression is regulated by methylation in other cell types and the exon array analysis identified their expression as induced by 5-aza-dC [52] [53]. Consistent with the exon array result, *stratifin* (*SFN*) mRNA levels were low or undetectable in all untreated fibroblasts (CAFs and control fibroblasts), and increased expression of *SFN* mRNA was detected in the majority of the 5-aza-dC treated fibroblasts (Fig. 2 A and B). We observed near complete methylation of the *SFN* promoter region in all CAFs by bisulfite sequencing (data not shown). Similarly, *TKTL1* mRNA levels were undetectable in almost all untreated fibroblasts except SC2 cells, and increased expression of *TKTL1* was detected in the majority of the 5-aza-dC treated fibroblasts (Fig. 2 C). Consistent with this, we found evidence of methylation of 5′ CpGs of *TKTL1* in fibroblasts by MSP (data not shown). Bisulfite sequencing of 18 CpG sites upstream of the *TKTL1* transcription start site (Fig. 2 D) revealed that all or most CpG sites were fully methylated in the non-expressing fibroblast lines, HPNE and SC3 and in CAF19 and CAF25 (Fig. 2 E). In contrast, the *TKTL1*-expressing line, SC2, lacked methylation of many of these sites, supporting a role for DNA methylation in the regulation of *TKTL1* expression. We also identified the upregulation of *DAZL* and *ANXA3* (data not shown), genes previously reported to be induced by 5-aza-dC [36], *NDN*, reported to be imprinted [54], and *MT1G*, reported to be methylated in other cancers [55].

We examined RNA profiles to determine if there were any differential responses to 5-aza-dc in CAFs compared to control fibroblasts. Thirty-five of the 42 genes induced in CAFs by 5-aza-dc (Table 1) were also induced in one or more of the control pancreatic fibroblast lines indicating that few if any genes are selectively and consistently upregulated by 5-aza-dC in CAFs. *DAZL* was the most highly induced gene in four CAFs (CAF12, CAF15, CAF16, and CAF19) and was among the top ten genes induced in two CAFs (CAF18 and CAF22) and one control fibroblast (HPNE) (data not shown). We performed hierarchical clustering of the genes induced by 5-aza-dC in CAFs and control fibroblasts to determine if there were observable differences in the overall patterns of gene response to 5-aza-dC, but there was no clustering by fibroblast class (Figure S3). Five of the seven CAFs clustered together, the other two CAFs clustering with the control fibroblasts.

Further evidence for this paucity of genes silenced by DNA methylation in CAFs comes from evaluating the genes underexpressed in CAFs. We identified genes that were underexpressed by a mean of ≥4.0-fold in all CAFs relative to pancreatic control fibroblasts (Table S2). Notably, there was no overlap between this list of 86 underexpressed genes and the genes induced by a mean of 2-fold by 5-aza-dc in CAFs (Table 1). These data indicate that few if any genes consistently underexpressed in CAFs are silenced by DNA methylation.

### Identification of candidate genes for hypomethylation analysis

We have previously identified 200 genes, such as *Smo*, upregulated in pancreatic CAFs relative to pancreatic control fibroblasts [27]. To identify candidate hypomethylated genes in CAFs, we merged this list of genes overexpressed in pancreatic CAFs with the list of 581 genes induced by ≥3.0 fold in at least one fibroblast cell line by 5-aza-dC treatment. This criterion identified 49 candidate genes (Table S1) some of which may be regulated by methylation, and potentially hypomethylated in pancreatic CAFs relative to control fibroblasts.

### 5-aza-dC treatment of human pancreatic CAFs, control fibroblasts and cancer cell lines

**Figure 3:**
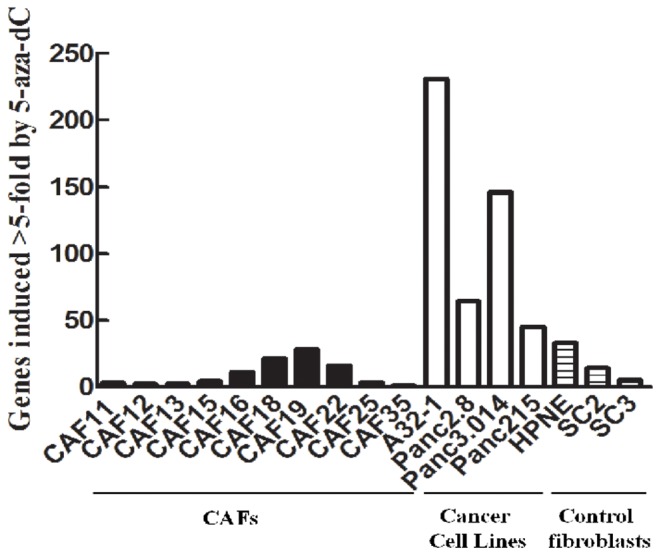
Number of genes induced by 5-aza-dC treatment in individual pancreatic CAFs, control fibroblasts and cell lines. An average of 123±86 genes were induced 5-fold or more by 5-aza-dC treatment in four pancreatic cancer cell lines, 9±10 genes in ten pancreatic CAFs (*P* = 0.0009) and 17±14 genes in three control pancreatic fibroblast lines.

We next compared the responses of CAFs and control fibroblasts to 5-aza-dC with the responses of pancreatic cancer cell lines. We generated an individual gene list for each fibroblast line or cell line and then counted the total number of genes induced by ≥5-fold in each line (Figure 3). We then compared the average number of genes induced by ≥5-fold by 5-aza-dC in the ten CAFs, the 3 control fibroblast lines, and the 4 pancreatic cancer cell lines. Significantly fewer genes were induced by 5-aza-dC in CAFs than in pancreatic cancer cell lines (*P* = 0.0009). The number of genes induced by ≥5-fold in the ten CAFs was only 9±10 genes (mean+/−standard deviation) compared to 17±14 genes induced by ≥5-fold in the three pancreatic control fibroblasts, and 123±86 genes induced by ≥5-fold in the 4 pancreatic cancer cell lines (Figure 3). There was no significant difference in the number of genes induced by ≥5.0 fold in CAFs and in pancreatic control fibroblasts. To ensure that we had selected a reasonable fold-change cutoff for comparison, we also compared the number of genes induced by ≥3-fold. Similar differences were also observed: The number of genes induced ≥3-fold by 5-aza-dC was 83±47 genes induced in control fibroblasts and 48±42 genes induced in CAFs and 430±271 genes in the pancreatic cancer cell lines (*P* = 0.0006 for CAFs relative to cancer cell lines). There were no significant differences related to patient age or gender in the number of genes induced by 5-aza-dC.

### Differential methylation of the candidate hypomethylated gene ADAM12

**Figure 4:**
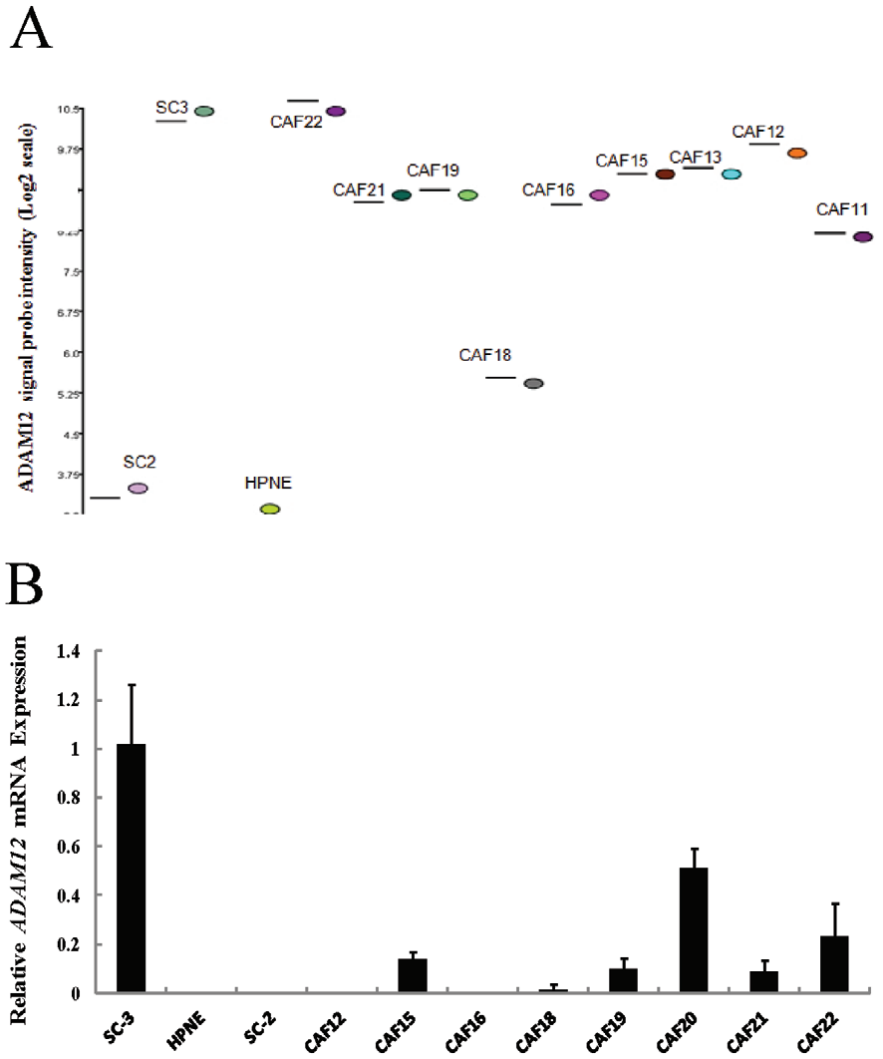
Analysis of *ADAM12* mRNA expression in pancreatic CAFs and control fibroblasts. (A) Affymetrix exon array analysis of *ADAM12* mRNA expression in pancreatic CAFs and control fibroblasts. (B) Quantitative RT-PCR analysis of *ADAM12* mRNA expression in pancreatic CAFs and control fibroblasts after normalization to *GAPDH* levels. Each assay was performed in triplicate. Data are means of three independent experiments; bars are SD values.

Our analysis of genes selectively upregulated by 5-aza-dC in CAFs yielded no genes known to be regulated by DNA methylation that influence stromal fibroblast behavior. We also examined our lists of genes differentially expressed in CAFs relative to control pancreatic fibroblasts for candidates that influence tumor/stromal interactions. One candidate overexpressed in CAFs was *ADAM12*. *ADAM12* (a disintegrin and metalloprotease 12), a regulator of cell-cell and cell-matrix interactions, is overexpressed in cancer-associated fibroblasts and is implicated in tumor progression [56]. We first confirmed upregulation of *ADAM12* mRNA in CAFs relative to control fibroblasts using quantitative RT-PCR. Consistent with our exon array result (Fig. 4a), *ADAM12* mRNA was highly overexpressed in the pancreatic control fibroblast derived from a pancreatitis specimen (SC3) and nine CAFs relative to the control fibroblasts HPNE and SC2 (Fig. 4b).

**Figure 5:**
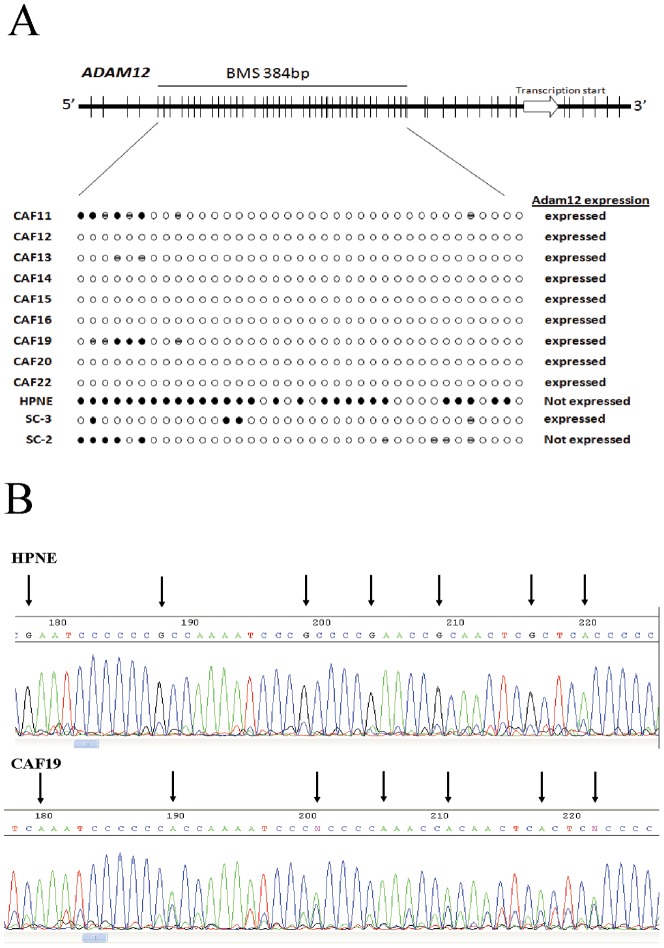
Bisulfite sequencing analysis of *ADAM12*. (A) Top: *ADAM12* gene structure and distribution of CpG dinucleotides. Short vertical bars represent CpG sites. Arrow points to transcriptional start site. Below: Bisulfite genomic sequencing analysis in pancreatic CAFs and control fibroblasts. Open circles represent unmethylated CpG sites, solid black circles methylated CpG sites, and hatched circles partially methylated CpG sites. (B) Bisulfite sequencing chromatograms of the *ADAM12* promoter in a pancreatic CAF (CAF19) and control fibroblast line (HPNE). Arrows point to cytosine residues.

To determine if *ADAM12* was differentially methylated in CAFs compared to control fibroblasts, we performed bisulfite sequencing of the *ADAM12* gene promoter in 9 CAFs and 3 control fibroblast lines. We amplified a 481-bp region upstream of the *ADAM12* transcription start site containing 38 CpG sites (Figure 5a). Bisulfite sequencing revealed that 29 of 38 (76%) CpG sites were fully methylated in the control fibroblast line HPNE (Figure 5b). By contrast, six of nine CAFs (CAF12, CAF14, CAF15, CAF16, CAF20, and CAF22) were completely (100%) unmethylated at all CpG sites. The remaining CAFs were partial methylated at 2 to 7 of 38 CpG sites and fully unmethylated at all other CpGs (Figure 5a). The control fibroblasts SC2 and SC3 were fully methylated at CpGs near the 5′ end of the sequenced region and partially methylated at CpGs near the 3′ end of this region. They were fully unmethylated at all other CpGs. Thus, there was relative hypomethylation at multiple CpGs between the fibroblasts that expressed *ADAM12* and those that did not, implicating aberrant hypomethylation of *ADAM12* as a mechanism for its overexpression in pancreatic CAFs compared to control pancreatic fibroblasts.

**Figure 6:**
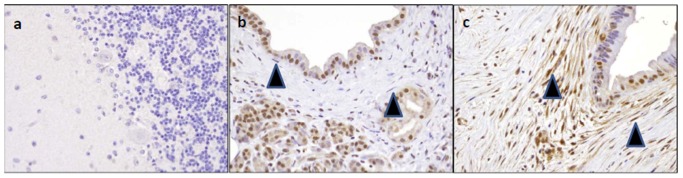
Immunohistochemical analysis of Adam12 protein expression in tissue microarrays. (A) Adam12 protein expression is undetectable in the granule cell layer of the brain (negative control tissue). (B) Stromal fibroblasts (arrows) surrounding normal pancreatic duct do not label Adam12. (C) Cancer associated fibroblasts (arrows) in a primary pancreatic adenocarcinoma are strongly positive for Adam12 protein; magnification, 20×.

To determine if Adam12 protein was overexpressed in the stroma of primary pancreatic adenocarcinomas, we performed immunohistochemistry on tissue microarrays. While Adam12 protein expression was not detected in fibroblasts surrounding the normal pancreatic duct (Figure 6b), CAFs of primary pancreatic adenocarcinomas were positive for Adam12 protein expression (Figure 6c). Adam12 expression was also observed in pancreatic epithelial cells and invasive pancreatic cancers.

## Discussion

Using a drug-based gene reactivation approach combined with global gene expression profiling, we find that human pancreatic CAFs are significantly less sensitive than cancer cell lines to 5-aza-dC-mediated gene expression changes. While an average of 134 genes were induced 5-fold or more by 5-aza-dC treatment of four human pancreatic cancer cell lines, an average of only 10 genes were induced in seven pancreatic CAFs. We confirmed 5-aza-dC-induced depletion of Dnmt1 protein and induction of genes known to be induced by 5-aza-dC. We also observed comparable Dnmt1 protein levels by Western blot or in *DNMT1* RNA levels by Exon array in pancreatic CAFs and cancer cell lines (data not shown), suggesting that the limited number of genes induced by 5-aza-dC was not due to a lack of *DNMT1* expression in CAFs or a lack of Dnmt1 inhibition by 5-aza-dC. This is consistent with the recent observation that hypomethylation in gastric CAFs could not be attributed to any difference in mRNA levels of *DNMT1*, *DNMT3a*, or *DNMT3b* between normal and cancer-associated myofibroblasts [36]. Overall, our results indicate that pancreatic CAFs do not undergo promoter DNA hypermethylation to the same extent as pancreatic cancer cells.

Our results are consistent with one previous report that 5-aza-dC-induced approximately twice as many genes (61 genes induced ≥4-fold) in a bladder cancer cell line (T24) than in normal fibroblasts (34 genes induced ≥4-fold) [45]. Similarly, we found an average of 17 genes was induced ≥5-fold in our pancreatic control fibroblasts. The 123 genes induced by 5-aza-dC in the 4 pancreatic cancer cells lines in this study was almost identical to the number of genes induced in 4 different pancreatic cancer cell lines (n = 131) in an earlier study by our group [30], [57]. This result is consistent with the hypothesis that CAFs are not subject to many promoter hypermethylation-induced gene silencing events compared to neoplastic epithelial cells. Previously, we demonstrated that 5-aza-dC treatment of the HPV E6E7-immortalized pancreatic non-neoplastic cell line, HPDE induced the expression of 93 genes (>5-fold with the Affymetrix U133 microarray) [30], [57], which is higher than the number of genes induced in fibroblasts but somewhat less than the number induced in pancreatic cancer cell lines. We also find that fibroblasts from non-neoplastic pancreata also have significantly fewer genes induced by 5-aza-dC than pancreatic cancer cell lines. These control fibroblasts respond similarly to 5-aza-dC as CAFs respond with respect to the numbers of genes.

Our findings indicate that despite residing in the same tumor microenvironment and undergoing similar environmental influences, it is the cancer cells that acquire most of the gene silencing DNA methylation alterations. Perhaps this is not surprising as CAFs are not thought to be under the same selective pressures as cancer cells. It was particularly notable that there was no evidence of silencing of tumor suppressor genes in CAFs supporting the notion that these cells are not under clonal selection [34]. In addition to clonal selection pressures, one of the proposed mechanisms for DNA methylation events in cancer cells are the mutations occurring during clonal expansion, some of which have been reported to influence DNA methylation. For example, amplifications in PML-RAR recruit DNA methyltransferases to promoters and cause hypermethylation [58]. Because CAFs do not undergo a similar clonal expansion from oncogenic and tumor suppressor mutations, it is not expected that they would acquire mutations that contribute to altered methylation patterns. Pancreatic and other cancer cells undergo methylation-induced silencing of numerous growth regulatory genes during clonal expansion (e.g. *p16*, *hMLH1*, *p14arf*, *SPARC*, *RELN*, *TFPI-2* and others [3], [30], [57], [59], [60], [61], [62]) and it is notable that we found no evidence that these genes are silenced by methylation in CAFs. In addition, age-related changes in DNA methylation did not appear to have had a major influence on fibroblast gene silencing. Although age-related DNA methylation are thought to be an important contributor to the methylation alterations of cancer cells such changes typically induce partial methylation of a small percentage of cells not sufficient to induce widespread gene silencing [63]. One explanation for the relative lack of methylation found in CAFs is the relative plasticity of fibroblasts as compared to epithelial cells. Studies comparing DNA methylation patterns at different states of differentiation indicate that cells undergo methylation-induced silencing of genes associated with pluripotency, development, and imprinting, suggesting that terminally differentiated cells would undergo more DNA methylation and would be more susceptible to 5-aza-dC induced reexpression of these genes [64], [65]. The plasticity of fibroblasts suggests that these cells are less differentiated than epithelial cells, consistent with the observation that they are less responsive than epithelial cells to 5-aza-dC treatment.

Our results suggest that few genes are silenced by DNA methylation in CAFs. Notably, 5-aza-dC does not reactivate the expression of all genes silenced by DNA methylation and works synergistically with other epigenetic modifying drugs such as histone deacetylase (HDAC) inhibitors, so it is possible that some genes are silenced by methylation in CAFs that are not induced by 5-aza-dC alone.

We selected *ADAM12* for further analysis because it was one of the few genes that we found to be overexpressed in pancreatic CAFs [27] and known to function in tumor-stromal cell interactions. We found relative hypomethylation of the *ADAM12* gene promoter in nine pancreatic CAFs expressing ADAM12 relative to the non-expressing fibroblast lines HPNE and SC2. Consistent with these findings, we observed overexpression of Adam12 protein in CAFs in primary pancreatic adenocarcinomas. Adam12 overexpression has not previously reported in pancreatic cancer stroma but is known to be overexpressed in cancer cells of multiple tumor types [66], and urinary Adam12 has been evaluated as a potential marker of bladder and urinary cancers [67].

Gene hypomethylation of overexpressed genes has been observed in pancreatic and other cancers [68] and promoter hypomethylation correlates with gene transcription [69]. Studies investigating mechanisms of demethylation have investigated whether such hypomethylation is passive rather than active [70], and recently, evidence has emerged implicating an active DNA demethylase function to DNA glycosylases [71].

The present results may hold important implications for therapeutic targeting of CAFs. Combined with previous studies indicating that fibroblasts are less susceptible than cancer cells to the cytotoxic effects of 5-aza-dC ([72] and our own observations), these data suggest that CAFs would not likely be very responsive to targeting w/DNA demethylating agents. They further suggest that using methylation markers in CAFs for diagnostic purposes may not be a viable approach, since CAFs do not undergo many DNA methylation alterations compared to cancer cells.

In conclusion, we find that treatment with the DNMT1 inhibitor 5-aza-dC induces remarkably fewer genes in pancreatic CAFs than pancreatic cancer cells. Although further studies are needed to clarify the extent of epigenetic changes that may contribute to the CAF phenotype, our observations suggest that attempts to target the stromal cells therapeutically may need to focus directly on genes mediating tumor-stromal cell interactions, rather than on targeting CAFs with DNA methylation inhibitors.

## Supporting Information

Figure S1 Hierarchical clustering of the global gene expression profiles of pancreatic fibroblasts.
(PPT)

Figure S2 The effect of 5-aza-dc on the proliferation of CAF19 cells as measured by MTT.
(PPTX)

Figure S3 Hierarchical clustering of the genes expression induced in pancreatic fibroblasts by 2-fold or more by 5-aza-dC.
(PPTX)

Table S1 Genes induced by 5-aza-dC and overexpressed in pancreatic CAFs relative to control fibroblasts.
(XLSX)

Table S2 Candidate hypermethylated genes underexpressed in CAFs relative to control fibroblasts.
(XLSX)
