# Supplementary figures and images for: Unlike Pancreatic Cancer Cells Pancreatic Cancer Associated Fibroblasts Display Minimal Gene Induction after 5-Aza-2′-Deoxycytidine
Source: PLoS One. 2012 Sep 11;7(9):e43456. doi: 10.1371/journal.pone.0043456 (PMC3439436; doi:10.1371/journal.pone.0043456)

## Slide 1
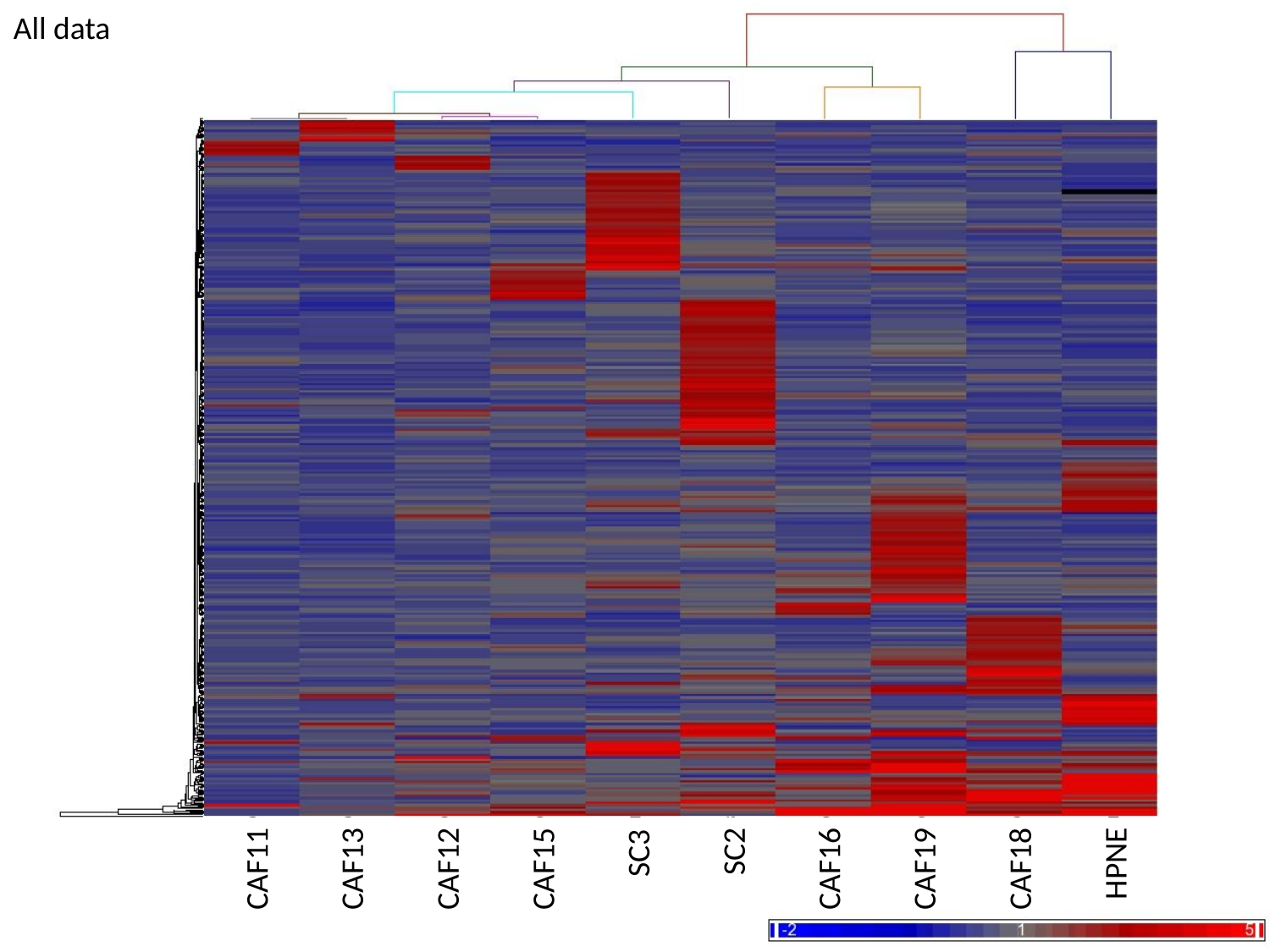

All data
SC2
SC3
HPNE
CAF11
CAF13
CAF12
CAF15
CAF16
CAF19
CAF18

Supplement: Figure S1 — Hierarchical clustering of the global gene expression profiles of pancreatic fibroblasts. (PPT) [file pone.0043456.s001.ppt]

## Slide 1
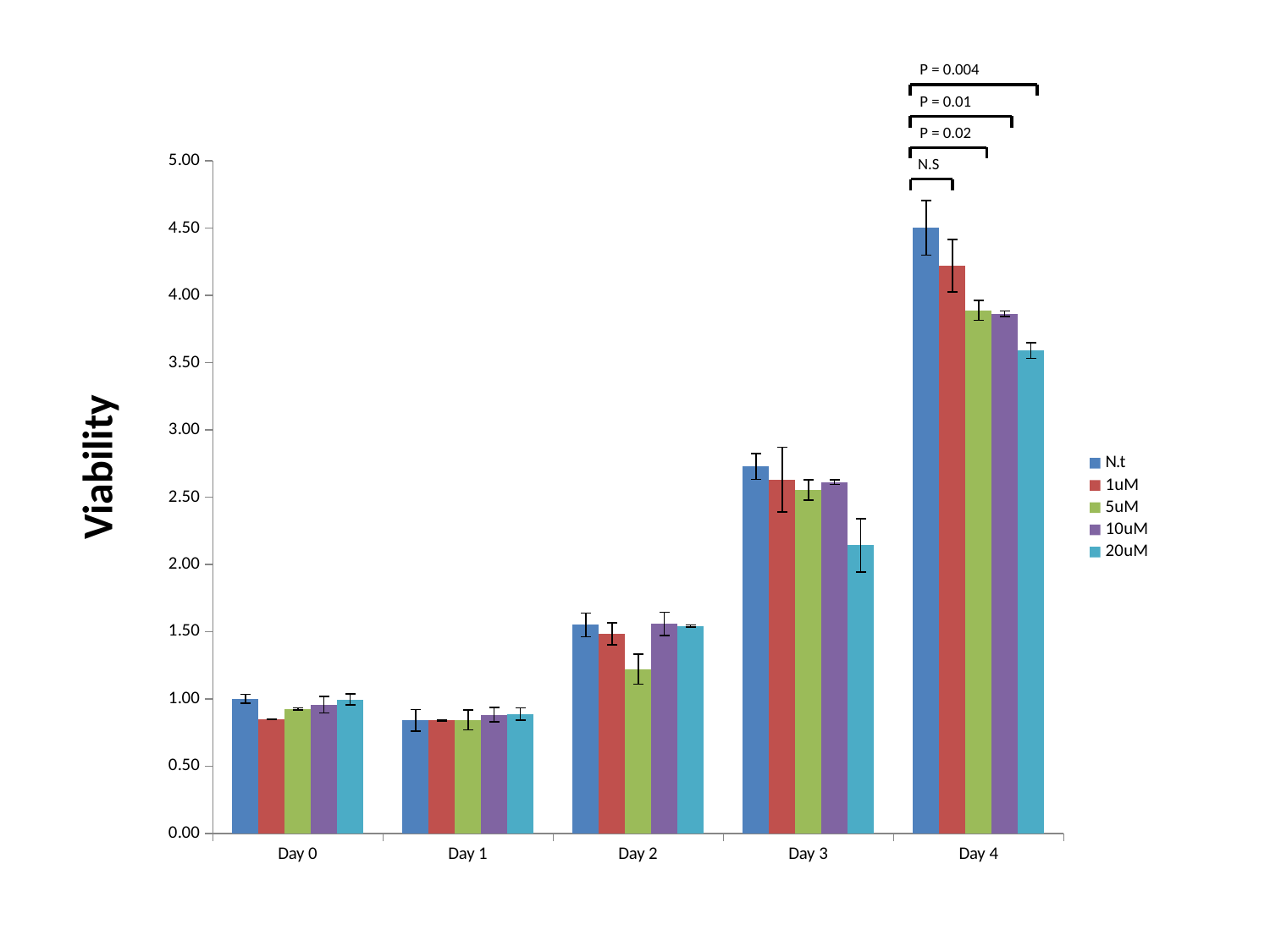

P = 0.004
P = 0.01
P = 0.02
### Chart
| Category | N.t | 1uM | 5uM | 10uM | 20uM |
|---|---|---|---|---|---|
| Day 0 | 1.0 | 0.8483063328424151 | 0.9248895434462443 | 0.9572901325478648 | 0.9955817378497789 |
| Day 1 | 0.8407321691563211 | 0.8407321691563214 | 0.8436776772564699 | 0.8819692825583842 | 0.8878602987586784 |
| Day 2 | 1.5499684409846406 | 1.483694508731326 | 1.2215442878182186 | 1.5573322112350092 | 1.5426046707342718 |
| Day 3 | 2.7275405007363798 | 2.630338733431518 | 2.553755522827687 | 2.61266568483063 | 2.141384388807071 |
| Day 4 | 4.503471491689457 | 4.222175468125388 | 3.889333052808752 | 3.8628234799074237 | 3.588891226593728 |N.S
Viability

Supplement: Figure S2 — The effect of 5-aza-dc on the proliferation of CAF19 cells as measured by MTT. (PPTX) [file pone.0043456.s002.pptx]

## Slide 1
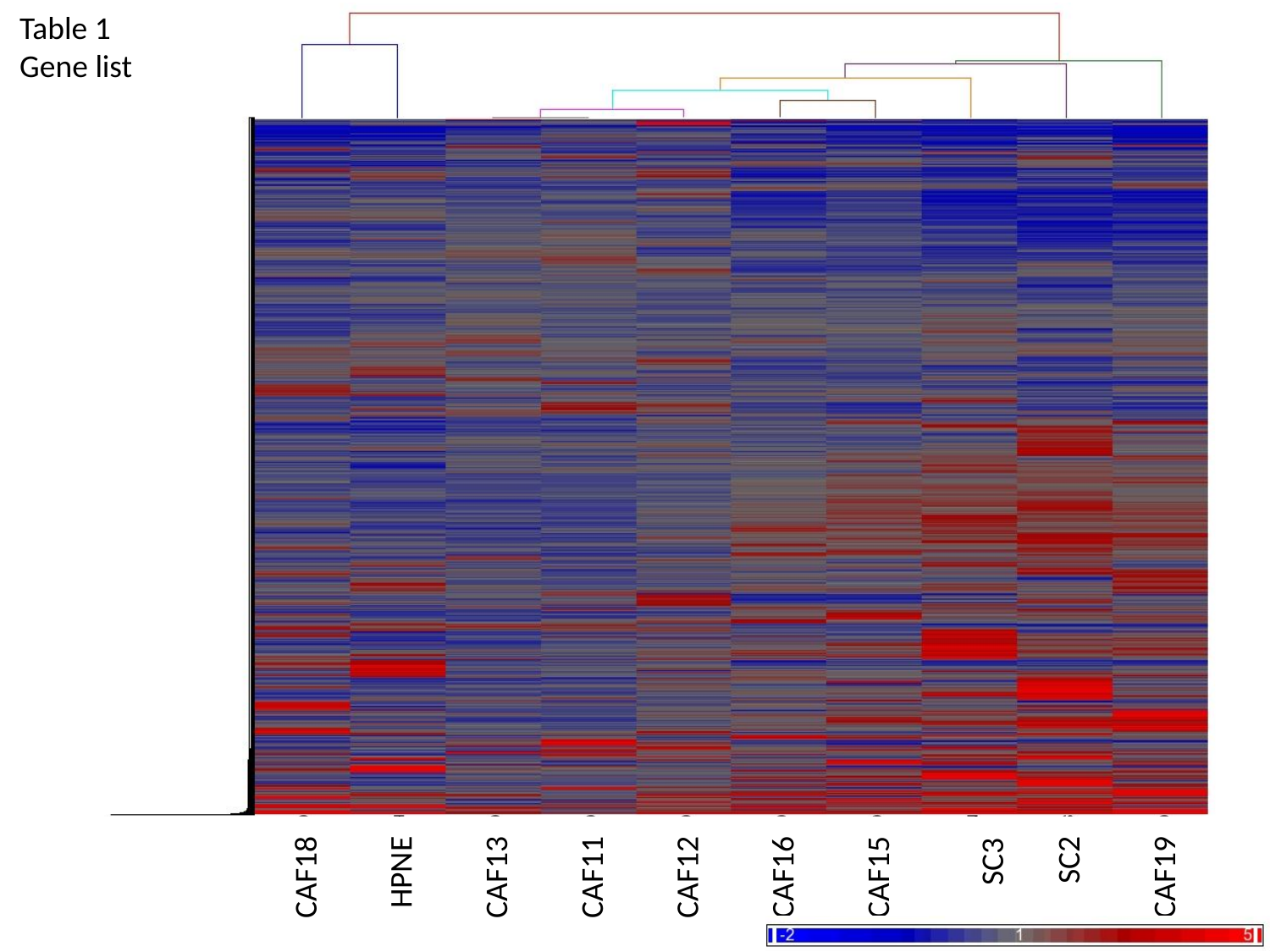

Table 1
Gene list
SC3
SC2
HPNE
CAF13
CAF12
CAF16
CAF18
CAF11
CAF15
CAF19

Supplement: Figure S3 — Hierarchical clustering of the genes expression induced in pancreatic fibroblasts by 2-fold or more by 5-aza-dC. (PPTX) [file pone.0043456.s003.pptx]
